# Supplementary material for: The expression of small RNAs in exosomes of follicular fluid altered in human polycystic ovarian syndrome
Source: PeerJ. 2020 Feb 19;8:e8640. doi: 10.7717/peerj.8640 (PMC7035867; doi:10.7717/peerj.8640)
Supplement: Supplemental Information 3 [file peerj-08-8640-s003.docx]

Fig 1C raw data
